# Mechanistic Insights into Succinic Acid as an Adjuvant for Ciprofloxacin in Treating *Pseudomonas aeruginosa* Growing Within Cystic Fibrosis Airway Mucus

**DOI:** 10.3390/microorganisms12122538

**Published:** 2024-12-09

**Authors:** Rosana Monteiro, Eduarda Silva, Maria Olivia Pereira, Ana Margarida Sousa

**Affiliations:** 1 Centre of Biological Engineering, LIBRO—Laboratório de Investigação em Biofilmes Rosário Oliveira, University of Minho, Campus de Gualtar, 4710-057 Braga, Portugal; rosana.monteiro@ceb.uminho.pt (R.M.); eduarda.silva@ceb.uminho.pt (E.S.); mopereira@deb.uminho.pt (M.O.P.); 2 LABBELS—Associate Laboratory, Braga/Guimarães, Portugal

**Keywords:** succinic acid, antibiotic adjuvant, *Pseudomonas aeruginosa*, ciprofloxacin, cystic fibrosis, small colony variants, mucoid phenotype, airway mucus, weak acid, organic acid

## Abstract

*Pseudomonas aeruginosa* is a major cause of chronic respiratory infections in patients with cystic fibrosis (CF), with biofilm formation contributing to its persistence and antibiotic resistance. This study aimed to gain insights into the mechanistic action of succinic acid as a ciprofloxacin adjuvant against clinically relevant CF isolates, including small colony variants and mucoid strains, and a ciprofloxacin-resistant strain grown within CF dense mucus. Time-kill assays in artificial CF mucus, along with planktonic and surface-attached biofilm experiments, were used to assess the activity of succinic acid alone and in combination with sublethal ciprofloxacin concentrations. Succinic acid demonstrated an adjuvant effect of ciprofloxacin against *P. aeruginosa* grown within CF mucus at pH levels below pKa1 during the early bacterial growth stages. In examining planktonic growth and biofilms under these conditions, we found that succinic acid demonstrated strong antibacterial and antibiofilm properties. Conversely, succinic acid activity decreased at later growth stages, though it enhanced the ciprofloxacin effect, especially against mucoid biofilms. Moreover, we noted that, in dense CF mucus, succinic acid activity was attenuated compared to a non-CF environment, indicating diffusion challenges. These findings underscore the potential of succinic acid as a therapeutic adjuvant for improving antibiotic treatment outcomes and overcoming biofilm-associated resistance in CF.

## 1. Introduction

Cystic fibrosis (CF) is a life-threatening genetic disorder caused by mutations in the cystic fibrosis transmembrane conductance regulator (CFTR) gene, resulting in the accumulation of thick, sticky mucus in various organs, most notably the lungs [1]. This abnormal mucus accumulation creates a favorable environment for persistent bacterial infections, which are a major cause of declining lung function, respiratory failure, and premature death in patients with CF. Among the pathogens responsible for these chronic respiratory infections, *Pseudomonas aeruginosa* is the most prevalent and concerning due to its strong association with prolonged hospitalizations and elevated mortality rates in patients with CF [2,3]. Once *P. aeruginosa* colonizes the lungs of patients with CF, the infection often progresses rapidly to chronic stages, becoming extremely difficult to eradicate, even with aggressive and prolonged antibiotic therapies [4,5].

The persistence of *P. aeruginosa* in CF airways is largely attributed to its ability to form biofilms, which are complex communities of bacteria encased in a protective self-produced extracellular matrix. These biofilms provide *P. aeruginosa* with a formidable defense against antibiotics, driven by mechanisms such as limited drug penetration, reduced metabolic activity, quorum sensing (QS), and the presence of persister cells [6]. Additionally, *P. aeruginosa* undergoes various adaptive changes, including the overproduction of alginate, leading to a mucoid phenotype, reduced motility, and the emergence of small colony variants (SCVs). These adaptations further enhance bacterial antibiotic tolerance, complicating treatment and facilitating long-term survival [4,5,7].

Even though therapeutic advances have significantly improved the life expectancy of patients with CF, the median age at death was 36.9 years in 2023 [2], and effective treatments targeting biofilm-associated infections remain elusive. Antibiotics, particularly ciprofloxacin, continue to be the cornerstone of CF infection management due to their potent bactericidal activity against planktonic bacteria. Ciprofloxacin exerts its action by inhibiting topoisomerase II (DNA gyrase) and topoisomerase IV, enzymes critical for DNA replication and transcription. However, like other antibiotics, ciprofloxacin is not specifically designed to target biofilms and, therefore, often fails in infection eradication [8,9]. Although some findings suggested that the biofilm matrix does not substantially impede ciprofloxacin penetration [10], decreased effectiveness across biofilm depth has been reported. This has been attributed to differential phenazine production, which influences bacteria’s metabolic activity and their susceptibility to ciprofloxacin [11]. Currently, no biofilm-targeting antibiotics are in the drug development pipeline, highlighting the urgent need for innovative strategies to disrupt biofilm formation and enhance the susceptibility of biofilm-resident bacteria to antibiotics.

One promising approach involves the use of adjuvants or helper compounds that sensitize or expose biofilm cells to antibiotics, thereby overcoming biofilm-associated resistance. Recently, Silva et al. [12] reported that succinic acid improved the efficacy of sublethal ciprofloxacin concentrations against *P. aeruginosa*, including antibiotic-resistant strains grown in CF artificial mucus. Succinic acid is a key metabolic intermediate in the tricarboxylic acid (TCA) cycle, playing a crucial role in energy production and metabolic reprogramming. As a naturally occurring weak organic acid, it is widely used across various industries, including the food, chemical, and pharmaceutical sectors [13].

Traditionally, organic acids, such as succinic acid, have been used as preservatives and disinfectants due to their ability to inhibit microbial growth [14,15]. Unlike antibiotics, which target specific bacterial components, organic acids exert their antimicrobial effects through multiple mechanisms. These include competition for energy, permeabilization of the bacterial outer membrane, increased intracellular osmotic pressure, and the disruption of biomolecule synthesis [16,17]. Furthermore, organic acids can reduce the local pH within the biofilm environment, destabilizing the extracellular polymeric substance (EPS) matrix and hindering biofilm development. They also interfere with QS, the bacterial communication system which regulates biofilm formation and maintenance, thereby inhibiting biofilm growth [18,19]. While these properties have been extensively studied in the context of food safety, surface disinfection, and topical and oral application [20,21,22], the therapeutic potential of organic acids as adjuvants in treating human biofilm-associated infections has only recently begun to be explored [23,24,25].

The ability of succinic acid to enhance ciprofloxacin efficacy, especially against resistant biofilms [12], along with being a naturally occurring substance in human metabolism and widely used in food and pharmaceuticals [26,27], positions it as an ideal candidate for further exploration as an antibiotic adjuvant, particularly in the context of biofilm-associated infections in patients with CF. The antimicrobial and antibiofilm effects of monoprotic and triprotic weak organic acids have been shown to vary significantly based on their pKa values and environmental factors such as dissolved oxygen and temperature [23,24,28,29]. However, it remains unclear whether similar principles apply to diprotic acids like succinic acid and whether this mechanism is observed against *P. aeruginosa* within CF mucus. Consequently, our study seeks to clarify the role of succinic acid as an adjuvant against clinically relevant CF isolates, including SCV and mucoid phenotypes. Specifically, our study sought to elucidate the mechanisms by which succinic acid acts on *P. aeruginosa* growing within CF airway mucus and understand how these mechanisms enhance ciprofloxacin activity. By addressing these questions, we aimed to provide new insights into the potential use of succinic acid as a therapeutic agent against CF biofilm-associated infections. The ultimate goal is to develop an alternative treatment that can effectively inhibit and eradicate *P. aeruginosa* biofilms within CF mucus. The broad-spectrum action and wide administration of ciprofloxacin extend the therapeutic advancements to several other infectious diseases, thus contributing to the global combat of antibiotic resistance.

## 2. Material and Methods

### 2.1. Bacterial Strains

Two *P. aeruginosa* clinical isolates from the lungs of patients with CF and one ciprofloxacin-resistant *P. aeruginosa* isolate from a patient without CF (referred to as PAI) were selected as a representative sample of the phenotypes routinely isolated from patients with CF. These strains were gently provided by different Portuguese hospitals [30]. The CF isolates exhibited two clinically relevant phenotypes: an SCV phenotype, designated as PA-SCV, and a mucoid phenotype, designated as PA-Muc. Bacterial cultures were routinely maintained in Tryptic Soy Broth (TSB, Liofilchem, Roseto degli Abruzzi, TE, Italy) or on Tryptic Soy Agar (TSA, Liofilchem, Roseto degli Abruzzi, TE, Italy) at 37 °C. To avoid potential adaptation to the laboratory environment, all strains were preserved in cryovials (Nalgene, Rochester, NY, USA) at −80 °C (±2 °C). Prior to each experiment, bacterial cells were grown overnight on TSA plates at 37 °C.

### 2.2. Ciprofloxacin Susceptibility Testing

The antimicrobial susceptibility of each *P. aeruginosa* strain was assessed using the disk diffusion method, following the guidelines provided in the NCCLS document M100-522 [31]. The cartridges of ciprofloxacin disks (5 μg) obtained from Liofilchem were stored at −20 °C and allowed to reach room temperature before use. Susceptibility testing was conducted by placing the disks on Mueller–Hinton agar (Liofilchem, Roseto degli Abruzzi, TE, Italy) plates inoculated with the bacterial strains, followed by incubation for 18–21 h at 37 °C. After incubation, the zones of inhibition were measured in millimeters (mm), and the strains were classified as resistant, intermediate, or sensitive based on the NCCLS criteria.

### 2.3. Preparation of Stock Solutions

Concentrated solutions of 440 mM succinic acid were prepared as described previously [12]. These solutions were used to achieve final concentrations of 20 mM or 50 mM in the bacterial cultures, as needed. Ciprofloxacin was purchased from Merck, and stock solutions of 1000 mg/L were prepared according to the manufacturer’s recommendations. All stock solutions were freshly prepared before the experiments.

### 2.4. Preparation of the Artificial CF Sputum Medium

Artificial sputum medium (ASM) was used to recapitulate the airway secretions of patients with CF, and it was prepared as described by Sriramulu et al. [32]. Briefly, ASM consisted of 5 g/L of mucin from pig stomach (Sigma-Aldrich, St. Louis, MO, USA), 4 g/L of DNA from salmon sperm (Sigma-Aldrich), 5.9 mg/L of diethylenetriaminepentaacetic acid (DTPA, Sigma-Aldrich), 5 g/L of NaCl, 2.2 g/L of KCl, and 5 g/L of casoamino acids (AMESRO) resuspended in distilled water. The pH was adjusted to 7 using Tris base. The medium was sterilized by autoclaving at 110 °C for 15 min. After cooling, 5 mL of egg yolk emulsion (Fluka, Buchs, Switzerland) was added, and the ASM was stored at 4 °C for up to one month.

### 2.5. ASM Assays

#### 2.5.1. Time-Kill Assays

Overnight cultures of each *P. aeruginosa* strain, grown in TSB at 37 °C, 120 rpm, were washed twice in sterile water by centrifugation at 9000× *g* for 5 min and diluted with sterile water to a final concentration of 4 × 10^9^ CFU/mL. Two milliliters of ASM was added to each well of a 24-well polystyrene plate (Orange, CA, USA), and 5 μL of the bacterial suspensions was inoculated on top, resulting in a final cell concentration of 1 × 10^7^ CFU/mL per well. ASM cultures were incubated aerobically at 37 °C under static conditions to simulate the reduced or absent ciliary movement observed in CF lungs [33,34]. Time-kill assays were conducted over 24 h to evaluate ciprofloxacin activity at 2 and 4 mg/L, with and without 20 mM of succinic acid from application at 0 h. Samples were collected at 4, 8, 12, and 24 h. At each time point, the content of the wells was aseptically collected and vigorously shaken to disrupt small aggregates or mucin-adherent cells. The resulting suspensions, representing the total bacterial populations in ASM, were plated on TSA to determine the number of culturable cells. Each experiment was performed independently five times.

#### 2.5.2. Impact of Bacterial Growth Stage on Treatment Outcome

To assess how the growth stage of *P. aeruginosa* affects treatment efficacy, bacteria were allowed to grow in ASM for 4, 12, or 24 h before being treated with 20 mM succinic acid, with or without 2 and 4 mg/L of ciprofloxacin. The effects of these treatments were determined 24 h after their application by quantifying the number of culturable cells as described above. Each experiment was performed independently five times.

#### 2.5.3. Effect of Acidic pH on Ciprofloxacin Efficacy

To investigate the effect of pH on the activity of ciprofloxacin, a concentrated solution of 2 M HCl, 44 mM citric acid, citrate buffer (pH 4), and phosphate citrate buffer (pH 2.2–2.4) was prepared. This solution was added to ASM at the start of incubation (0 h) to achieve a final pH of approximately 4. Further, ASM was inoculated as previously described, and, after 24 h of application of the acidic solutions, the number of culturable cells was determined. All stock solutions were freshly prepared before application. Experiments were conducted in triplicate.

#### 2.5.4. Inhibition of Planktonic Growth and Biofilm Formation Assay

Overnight cultures of each *P. aeruginosa* strain were grown in TSB at 37 °C, 120 rpm. The cultures were then washed with sterile water and diluted in TSB to a final concentration of 1 × 10^7^ CFU/mL. Diluted bacterial suspensions (2 mL per well) were added to 24-well plates and treated with 20 mM or 50 mM succinic acid, with or without 2 or 4 mg/L ciprofloxacin, at 0, 4, 12, and 24 h after biofilm formation. The treated bacterial cultures were incubated at 37 °C under static conditions. After 24 h of each treatment, the liquid content of the wells was carefully collected to quantify the number of culturable cells at the planktonic stage. The adhered biomass to the well surfaces was washed twice with sterile water to remove loosely attached cells. Subsequently, 2 mL of methanol was added to each well and left for 15 min to fix the biofilm biomass. The methanol was then discarded, and the plates were air-dried at room temperature. The biofilms were stained by adding 2 mL of 1% (*v*/*v*) crystal violet (CV) to each well and incubating for 5 min. Excess stain was removed by washing the wells twice with distilled water. To quantify the biofilm biomass, the CV was solubilized in 2 mL of 33% (*v*/*v*) acetic acid, and the absorbance was measured at 570 nm using a microplate reader (BioTek Synergy HT, Izasa, Alcobendas, Spain).

#### 2.5.5. Confocal Laser Scanning Microscopy

Biofilms were developed and treated on 13 mm plastic coverslips (Thermo Scientific, Waltham, MA, USA) placed inside 24-well polystyrene microtiter plates with TSB and incubated at 37 °C, following the procedure previously described. After the incubation period, the biofilms were rinsed twice with 0.9% (*w*/*v*) NaCl solution to remove non-adherent cells, and their viability was assessed using the LIVE/DEAD™ BacLight™ Bacterial Viability Kit (Invitrogen, Thermo Fisher Scientific, Waltham, MA, USA), which includes SYTO 9 and propidium iodide (PI) stains. Imaging of the biofilms was performed with an Olympus™ Fluo-View FV1000 confocal laser scanning microscope (CLSM), utilizing 10× and 40× objectives. SYTO 9-stained live cells were visualized with an excitation wavelength of 485 nm and an emission at 498 nm, while dead cells stained with PI were detected using an excitation wavelength of 536 nm and an emission at 617 nm. Each experiment was conducted in duplicate.

### 2.6. Statistical Analyses

Statistical analyses were conducted using Graph Pad Prism (version 9.5.1). Differences between treatments were evaluated using a one-way analysis of variance (ANOVA), followed by Tukey’s multiple comparisons test to assess significance at *p* < 0.05. Data were presented as mean ± standard error of the mean (SEM), and each experiment was performed independently at least three times.

## 3. Results

### 3.1. P. aeruginosa Susceptibility to Ciprofloxacin

*P. aeruginosa* susceptibility to ciprofloxacin was assessed using the disk diffusion method. PA-Muc and PA-SCV strains were sensitive to ciprofloxacin, whereas the PAI strain was resistant. This variation in susceptibility allowed us to evaluate the activity of succinic acid across different *P. aeruginosa* phenotypes and its potential role as a ciprofloxacin adjuvant against bacteria with distinct antibiotic susceptibilities. Due to the limited efficacy of ciprofloxacin at sublethal concentrations against *P. aeruginosa* in ASM [5,12], minimum inhibitory concentration (MIC) determination was deemed unnecessary for selecting effective dosages. Rather, referencing past research [12], sublethal ciprofloxacin concentrations of 2 and 4 mg/L were selected; although they had been previously demonstrated to be ineffective in ASM alone, they exhibited therapeutic potential when combined with succinic acid adjuvant.

### 3.2. Time-Kill Assays Reveal Enhanced Activity of Succinic Acid–Ciprofloxacin Combinations

**Figure 1:**
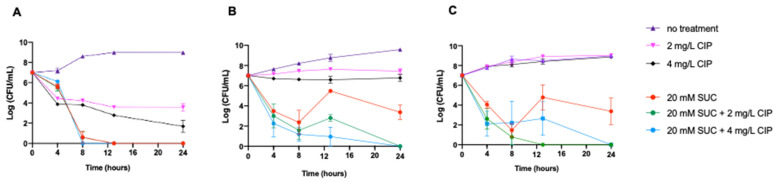
Time-kill curve for single-drug and two-drug combinations against *P. aeruginosa* over 24 h in ASM. (**A**) PA-Muc and (**B**) PA-SCV CF isolates and (**C**) PAI treated with 20 mM of succinic acid (SUC) with and without 2 and 4 mg/L of ciprofloxacin (CIP). All experiments were conducted independently at least five times. Data are presented as mean  ±  SEM.

To determine the combined effect of succinic acid and ciprofloxacin on *P. aeruginosa*, we conducted time-kill assays in ASM using the selected ciprofloxacin concentrations of 2 and 4 mg/L alone and in combination with 20 mM succinic acid. The time-kill assays revealed that ciprofloxacin alone was insufficient to achieve complete bacterial eradication across all strains (Figure 1), reinforcing the need for adjuvant therapies. However, when combined with 20 mM succinic acid, both concentrations led to significant reductions in culturable cell counts for all *P. aeruginosa* strains. Notably, bacterial growth was significantly suppressed within 8 to 12 h of combined treatment, with no culturable cells detected by 24 h. These findings underscored the potential of succinic acid as an effective adjuvant, prompting further investigation into its mechanisms of action in the CF airway context.

### 3.3. Absence of pH-Dependent Enhancement of Ciprofloxacin

Although some findings indicate that acidic conditions may impair the effectiveness of ciprofloxacin against pathogens, including *P. aeruginosa* [35], other evidence suggests that its efficacy may not be diminished under acidic conditions [36], pointing to a complex interaction with environmental pH, with efficacy variably affected by species or strain. In our study, the addition of succinic acid lowered the pH of ASM to approximately 4 over 24 h, yet enhanced ciprofloxacin action (Figure 1). This observation led us to hypothesize that succinic acid’s potentiating effect could be related to an acidic pH. To test this, we adjusted the pH of ASM using various solutions and evaluated their adjuvant effects on ciprofloxacin. Specifically, we used 19 mM HCl (pH 4.06  ±  0.10), 4 mM citric acid (pH 4.23  ±  0.04), citrate buffer (pH 4.62  ±  0.05), and phosphate-citrate buffer (pH 4.41  ±  0.03). We acknowledge that testing strong acids (e.g., HCl) or buffers may not be physiologically relevant in the context of CF lung therapeutics, but this approach allowed us to manipulate pH in a controlled manner, isolating it as a variable separate from any bioactive effects of succinic acid.

Our results indicated that none of these pH adjustments alone significantly impacted *P. aeruginosa* growth or enhanced ciprofloxacin’s killing efficacy (Figure S1 in Supplementary Materials). This contrasted with the potentiating effect of succinic acid observed earlier (Figure 1), suggesting that acidic pH alone did not account for the enhanced antibiotic activity.

### 3.4. Activity of Succinic Acid Dependent on pKa Values and Bacterial Growth Stage

Succinic acid is a weak organic diprotic acid with pKa values of 4.207 (pKa1) and 5.635 (pKa2) (source: PubChem, ID: 1110), and similar to other weak organic acids, its biological activity in ASM may be influenced by its ionization state, which is dependent on pH relative to its pKa values [23]. To investigate this hypothesis, we tested various concentrations of succinic acid corresponding to pH levels above and below pKa1 and assessed their effect on *P. aeruginosa* growth and ciprofloxacin efficacy.

**Figure 2:**
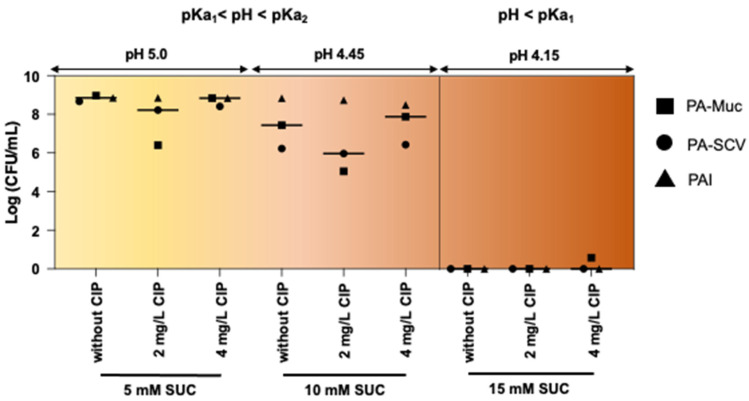
Comparison of succinic acid (SUC) activity against *P. aeruginosa* PA-Muc (square), PA-SCV (circle), and PAI (triangle) at pH levels below pKa1 (corresponding to 15 mM) and above pKa1 (corresponding to 5 and 10 mM), with and without 2 and 4 mg/L ciprofloxacin (CIP), after 24 h in ASM. Each experiment was performed independently two times. Geometric forms present individual means, and the line presents the median of the three bacterial strains.

Our results demonstrated a distinct pattern of activity according to the pH of ASM relative to the pKa values of succinic acid (Figure 2). When the pH was lower than pKa1, succinic acid appeared to eradicate *P. aeruginosa* from ASM, which diminished the need for ciprofloxacin enhancement. In contrast, at pH levels between pKa1 and pKa2, succinic acid’s antibacterial effect was markedly reduced, and its ability to potentiate ciprofloxacin was lost.

**Figure 3:**
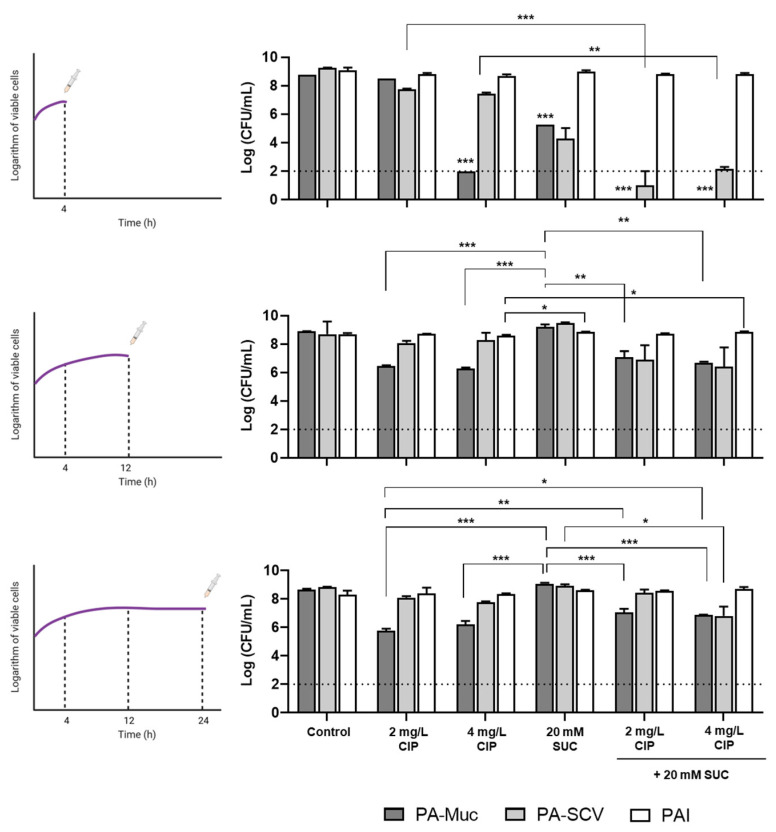
Effect of bacterial growth stage on succinic acid (SUC) activity and its interaction with 2 and 4 mg/L of ciprofloxacin (CIP) against *P. aeruginosa* Pa-Muc, PA-SCV, and PAI when applied after 4, 12, and 24 h in ASM cultures. All experiments were performed independently two times. Data are presented as mean  ±  SEM. One-way ANOVA analysis was used to compare the difference between the treated samples and the corresponding negative controls (* *p*  <  0.05, ** *p*  <  0.01, and *** *p*  <  0.001). Dash lines indicate the detection limit. (Figure partially created with BioRender https://www.biorender.com/ accessed on September 2024).

To further explore the antibacterial action and adjuvant effect of succinic acid, we evaluated its effects on *P. aeruginosa* at different growth stages by treating ASM cultures with 20 mM succinic acid (pH < pKa1) after 4, 12, and 24 h of growth. Our results showed that succinic acid’s antibacterial and adjuvant effects were reduced when applied at later stages of growth (Figure 3). However, we noted its consistent adjuvant effect in enhancing ciprofloxacin action against PA-Muc and PA-SCV when applied after 4 h of growth, despite its limited antibacterial activity when acting alone.

### 3.5. Succinic Acid Action Against Planktonic Cells and Biofilms

The previous experiments examined the entire *P. aeruginosa* population in ASM, encompassing both planktonic cells and suspended biofilms. To gain a clearer understanding of how succinic acid acts on bacteria and enhances ciprofloxacin efficacy, we recognized the need to investigate its effects on planktonic cells and biofilms separately. Due to technical challenges in physically separating these populations in ASM, we modified our experimental setup using a 24-well plate in vitro model for surface-attached biofilm formation.

In these assays, TSB with a baseline pH of 7.05  ±  0.05 was used. After adding 20 mM of succinic acid, the pH of TSB was lowered to 5.01. Based on our earlier findings (Figure 2), we hypothesized that increased concentrations of succinic acid would be necessary to reduce the pH below pKa1 and significantly affect *P. aeruginosa* growth and potentiate ciprofloxacin action. At a pH of 5.01 (above pKa1), neither succinic acid alone nor its combination with ciprofloxacin exhibited antibacterial activity across all three *P. aeruginosa* strains (Figure S2A–C, Supplementary Materials). However, succinic acid alone and when co-administered with ciprofloxacin effectively inhibited biofilm formation for all *P. aeruginosa* strains (Figure S2D–F, Supplementary Materials). Succinic acid alone inhibited biofilm establishment, but the synergistic effect with ciprofloxacin yielded complete biofilm inhibition.

**Figure 4:**
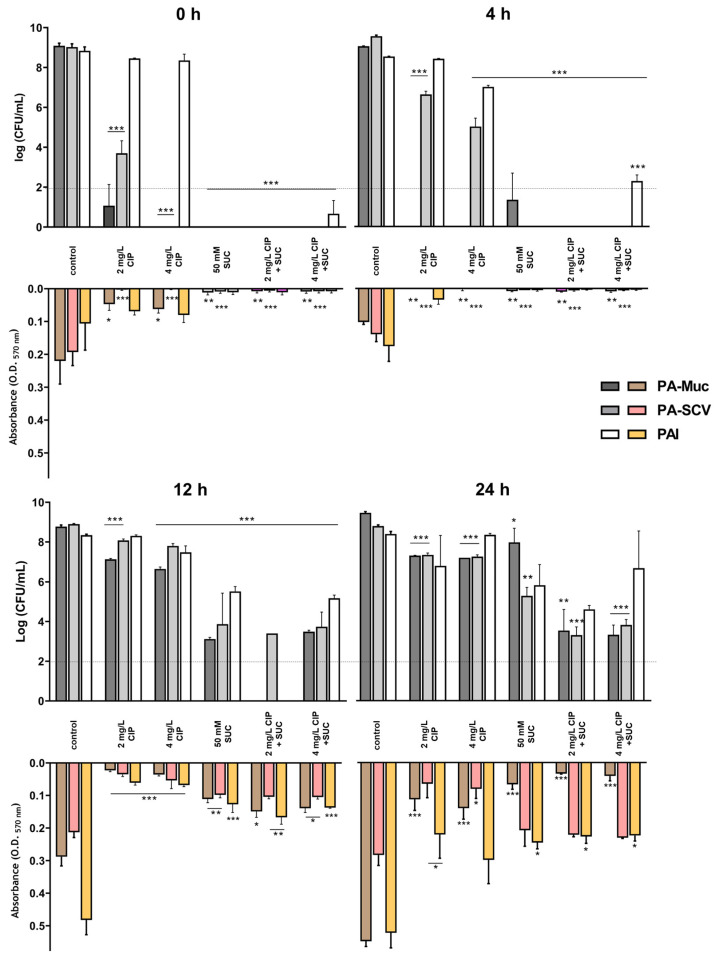
Effect of bacterial growth stage on succinic acid (SUC) activity and its interaction with 2 and 4 mg/L of ciprofloxacin (CIP) against *P. aeruginosa* Pa-Muc, PA-SCV, and PAI when applied at 0, 4, 12, and 24 h of planktonic growth (determined by CFU counting) and biofilm formation (determined by CV staining, measuring the O.D at 570 nm) in TSB. All experiments were performed independently at least three times. Values are the mean  ±  SEM. A one-way ANOVA analysis was used to compare the difference between the treated samples and the corresponding negative controls (* *p*  <  0.05, ** *p*  <  0.01, and *** *p*  <  0.001). Dash lines indicate the detection limit.

To further investigate pH-dependent effects below pKa, we tested 50 mM succinic acid (pH 4.15) across different bacterial growth phases (0, 4, 12, and 24 h). The antimicrobial and antibiofilm effects of succinic acid were most prominent at early growth stages (0 and 4 h), leaving little room for improvement in ciprofloxacin action (Figure 4). Succinic acid at 50 mM, with pH 4.15 below pKa1, exhibited antibacterial activity not observed previously (Figure S2A–C, Supplementary Materials), supporting a mechanistic action dependent on its pKa values, as observed earlier (Figure 2).

At later stages (12 and 24 h), succinic acid’s bactericidal activity diminished against planktonic bacteria, though it continued to improve ciprofloxacin action, achieving approximately a 5-log reduction in bacterial load for PA-Muc and PA-SCV. Regarding mature biofilms, succinic acid alone exhibited similar antibiofilm activity to ciprofloxacin on 24 h old biofilms, with synergy observed only against PA-Muc biofilms. This reduced efficacy against planktonic bacteria and mature biofilms likely explains the limited success observed in the ASM in vitro model at later stages of bacterial growth (Figure 3).

### 3.6. Antibiofilm Activity Assessed by CLSM

**Figure 5:**
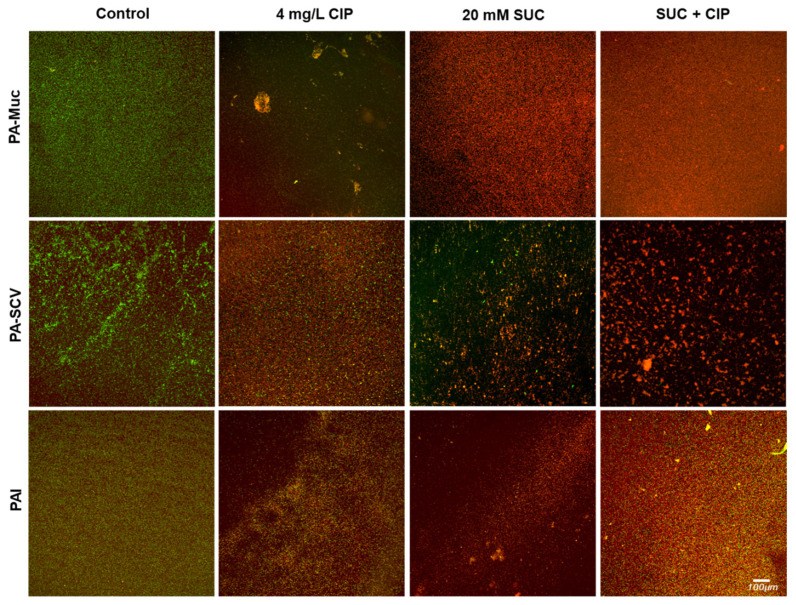
Confocal microscopy of 24 h old *P. aeruginosa* biofilms treated with 4 mg/L of ciprofloxacin (CIP), 20 mM of succinic acid (SUC), and 4 mg/L of ciprofloxacin (CIP) plus 20 mM of succinic acid (SUC + CIP). Biofilms were stained with the LIVE/DEAD™ kit and live and dead cells can be seen in green and red, respectively. The images were acquired using an objective of 10×.

CV staining is a quantitative method for assessing biofilm biomass, but it does not differentiate between the biofilm matrix and bacterial cells. Therefore, CLSM was used to visualize the effects of succinic acid on both the structure of mature 24 h old biofilms and cell viability. CLSM image analysis showed that treatment with succinic acid alone primarily led to bacterial cell death (Figure 5), whereas a significant fraction of bacteria remained viable following ciprofloxacin treatment. The combined effects of succinic acid and ciprofloxacin appeared to be strain-dependent. For PA-Muc biofilms, no synergy was observed, as the combined treatment yielded results similar to succinic acid alone. In contrast, for PA-SCV biofilms, complete bacterial eradication was achieved when both agents were used together, suggesting a potential synergistic effect. In PAI biofilms, succinic acid alone was more effective than the combination, suggesting an antagonistic interaction. Viable cells were detected after the application of both agents in comparison with succinic acid alone.

These findings suggest that succinic acid exerted effective antibiofilm activity against mature biofilms, likely by penetrating the biofilm matrix and exerting its bactericidal effects. However, the thin and widely spread structure of these *P. aeruginosa* biofilms may have facilitated succinic acid penetration into the matrix, enhancing its effectiveness.

## 4. Discussion

Our results supported succinic acid as a promising adjuvant candidate that may address the urgent need for more effective treatments against biofilm-associated infections in CF [12]. Importantly, our findings offered new insights into the mechanisms by which succinic acid enhances ciprofloxacin activity against biofilm surrounded by dense airway mucus.

Succinic acid exhibited strong bactericidal activity against *P. aeruginosa* grown in CF mucus at pH levels below its pKa1 value (4.2), indicating that its adjuvant effects may depend on environmental pH. This can be attributed to the fact that, at pH values below pKa1, succinic acid remains largely undissociated, allowing its neutral form to freely cross bacterial membranes. Once internalized, it dissociates in the cytoplasm (where the pH is around 7), releasing acid ions (ROO^−^) and protons (H^+^). This process leads to cytoplasmic acidification and a collapse of intracellular pH homeostasis. Bacteria are then forced to actively expel H^+^ ions through transport mechanisms to maintain the internal pH, consuming large amounts of energy [17]. During the early growth stages, planktonic cells are metabolically active, a significant portion of cellular energy is devoted to essential processes, and they are not able to spend a lot of energy maintaining intracellular pH homeostasis. Consequently, the disruption of pH balance leads to a collapse of intracellular homeostasis, causing multiple cellular dysfunctions [37]. This includes reduced enzyme activity, protein denaturation, and damage to membranes or DNA, ultimately leading to cell death [38,39,40,41]. Moreover, by acidifying the local environment, succinic acid can interfere with the signals and conditions that promote biofilm formation, making it harder for bacteria to establish mature biofilms [18,19,42]. 

Despite succinic acid showing promise, *P. aeruginosa* embedded in ASM can survive succinic acid at early stages of growth. In this environment, bacteria are surrounded by a thick mucus layer that acts as an additional physical barrier, hindering the diffusion and efficacy of succinic acid. The dense and viscous nature of this CF mucus creates a network that can slow or even block the efficient delivery of drugs, often resulting in treatment failure, bacterial survival, and the persistence of infection [5,12,43,44,45]. While succinic acid alone does not lead to *P. aeruginosa* death in ASM, the induced acidification could cause changes in the bacterial cell membrane, including increased membrane fluidity and permeability, which may facilitate the uptake of ciprofloxacin [46,47]. Moreover, high concentrations of succinic acid can cause metabolic imbalances, leading to the accumulation of certain metabolites and reduced ATP production. Metabolically stressed cells are less capable of activating defense mechanisms, such as efflux pumps, which can expel antibiotics, thereby increasing ciprofloxacin’s efficacy [48,49]. Succinic acid can also disrupt essential cellular processes, including DNA repair and enzymatic function, rendering bacterial cells more susceptible to ciprofloxacin, as weakened cells are less able to counteract the antibiotic’s effects. Together, these mechanisms may underscore succinic acid’s potential to boost ciprofloxacin action, particularly against resistant CF phenotypes. The effectiveness of succinic acid alone against SCV and mucoid *P. aeruginosa* is of utmost clinical relevance. SCV and mucoid *P. aeruginosa* strains are known for their robust biofilm formation and resistance to antibiotics, often leading to chronic, difficult-to-treat infections in patients with CF [50,51,52,53,54]. Thus, the activity of succinic acid against these phenotypes suggests potential therapeutic benefits.

Ciprofloxacin exhibits poor aqueous solubility, and salt formation, using suitable counterions, has been demonstrated to significantly enhance its solubility. For instance, Paluch et al. (2013) [55] showed that succinic acid forms both crystalline and amorphous salts, with ciprofloxacin achieving a more than 300-fold increase in water solubility. Similarly, Hibbard et al. (2023) [56] studied dicarboxylic acid salts of ciprofloxacin, including those formed with succinic acid, and they confirmed proton transfer between ciprofloxacin and the acid counterions, which contributed to enhanced solubility and antibacterial activity in planktonic bacteria. Our study did not explicitly investigate the chemical interactions between ciprofloxacin and succinic acid in ASM, and, therefore, the possibility of succinic acid enhancing ciprofloxacin solubility through salt formation cannot be ruled out and should be addressed in future investigations.

At later growth stages, the effects of succinic acid alone are notably reduced, primarily exhibiting weak antibacterial properties and the partial dismantling of mature biofilms. Consequently, its efficacy as an adjuvant diminishes. The survival of planktonic *P. aeruginosa* at these stages may be attributed to the stationary phase, characterized by significantly lower metabolic activity. In this phase, several of the key metabolic processes targeted by weak acids are downregulated, such as DNA replication, protein synthesis, and cell division, reducing the cells’ susceptibility to succinic acid’s effects. Moreover, *P. aeruginosa* cells in the stationary phase are known to upregulate specific stress-response mechanisms, including acid resistance pathways. These mechanisms may include the increased expression of proton pumps, including F1F0-ATPase which actively expels protons from the cell to maintain pH balance [57,58,59], as well as the accumulation of protective molecules, such as polyamines (e.g., spermidine), amino acids (e.g., glutamate), and other small molecules which can buffer intracellular pH changes [57,60,61,62]. Structural changes in the cell membrane may also occur, making it less permeable to weak acids, further preventing intracellular acidification. This can be triggered by the stationary growth stage as well as by growing in a complex environment such as ASM [5,63]. Consequently, when succinic acid acidifies the cytoplasm, these enhanced mechanisms can mitigate the impact by either pumping out the excess protons or neutralizing them, preventing the lethal acidification of the intracellular environment.

Mature biofilms are notoriously difficult to treat due to their dense clusters of bacteria embedded within a protective EPS matrix, exhibiting phenotypic and genotypic heterogeneity. Additionally, bacteria within biofilms communicate via QS to coordinate collective responses to stress [64,65,66]. Despite the challenges in eradicating mature biofilms using succinic acid alone, 24 h old mucoid biofilms seemed particularly susceptible to succinic acid alone and combined with ciprofloxacin. Initially, our findings suggested that succinic acid dismantled biofilms by degrading components of the EPS matrix due to a significant decrease in biofilm biomass. Kundukad et al. [23] previously reported a similar effect on mucoid *P. aeruginosa* biofilms using citric acid below pKa1. However, microscopic observations of the biofilms treated with succinic acid revealed no significant decrease in biofilm biomass, suggesting instead that succinic acid penetrated the biofilm matrix to exert its bactericidal effects directly. This may be facilitated by the relatively thin structure of the biofilms, which could allow for greater penetration. Moreover, microscopic observations confirmed this mechanist action in other *P. aeruginosa* biofilms.

Despite the promising results, our study has limitations that require further investigation. The reduced efficacy of succinic acid in a dense CF mucus environment indicated that its clinical application may require optimized delivery strategies to ensure adequate penetration and sustained activity. Additionally, although the in vitro CF model used in this study provides a useful representation of the in vivo scenario, it cannot fully replicate the physiological conditions in CF lungs, including immune responses and variable mucus densities. Comparative studies with other organic acids or adjuvants could also shed light on specific mechanisms and help identify the most effective adjuvant candidates for CF-related biofilm infections.

## 5. Conclusions

This study elucidated the mechanistic role of succinic acid as an adjuvant to ciprofloxacin against *P. aeruginosa* growing within CF mucus, highlighting its efficacy against clinically relevant CF strains, including SCVs, mucoid, and ciprofloxacin-resistant phenotypes. Our findings showed that its antibacterial and antibiofilm effects were closely tied to its pKa values and the bacterial growth stage. At pH levels below pKa1, succinic acid exhibited potent bactericidal and antibiofilm activity, particularly during early bacterial growth, against SCVs, mucoid, and ciprofloxacin-resistant strains. Additionally, succinic acid enhanced ciprofloxacin efficacy even at sublethal concentrations, likely by increasing bacterial membrane permeability and disrupting energy metabolism. Despite these results being promising, some limitations of succinic acid’s action were observed, including the reduced efficacy at pH levels above pKa1, later bacterial growth stages, and the presence of dense CF mucus. The complex environment of CF airway secretions, represented by ASM, revealed that mucus can act as a physical barrier, hindering the diffusion of succinic acid and impacting its biological activity and ability to enhance ciprofloxacin action. Nevertheless, succinic acid still enhanced the action of ciprofloxacin against *P. aeruginosa* by overcoming multiple aspects of CF disease and bacterial physiology. The ability of succinic acid to act on multiple fronts, including antibacterial and biofilm inhibition, makes it a promising candidate for further development as a therapeutic adjuvant, potentially filling a critical gap in the treatment of biofilm-associated infections in patients with CF.

## Data Availability

The original contributions presented in this study are included in the article/supplementary material. Further inquiries can be directed to the corresponding author.

## Supplementary Materials

The following supporting information can be downloaded at: https://www.mdpi.com/article/10.3390/microorganisms12122538/s1, Figure S1: Effects of pH 4, adjusted using HCl, citric acid, citrate buffer, and phosphate buffer, on P. aeruginosa growth in ASM after 24 h. (A) PA-Muc and (B) PA-SCV CF isolates and (B) PAI. Each experiment was performed independently at least two times. Data are presented as mean ± SEM. Dash lines indicate the detection limit; Figure S2: Effect of 20 mM of succinic acid (SUC) activity and its interaction with 2 and 4 mg/L of ciprofloxacin (CIP) against P. aeruginosa (A,D) Pa-Muc, (B,E) PA-SCV and (C,F) PAI when applied at 0 h on (A,B) planktonic growth and (E,F) biofilm formation in TSB. All experiments were performed independently at least three times. Values are the mean ± standard deviation.
