# Supplementary material for: Mechanistic Insights into Succinic Acid as an Adjuvant for Ciprofloxacin in Treating Pseudomonas aeruginosa Growing Within Cystic Fibrosis Airway Mucus
Source: Microorganisms. 2024 Dec 9;12(12):2538. doi: 10.3390/microorganisms12122538 (PMC11678660; doi:10.3390/microorganisms12122538)
Supplement: Supplementary file 1 [file microorganisms-12-02538-s001.zip › microorganisms-3318232-supplementary.pdf]

Supplemental Material

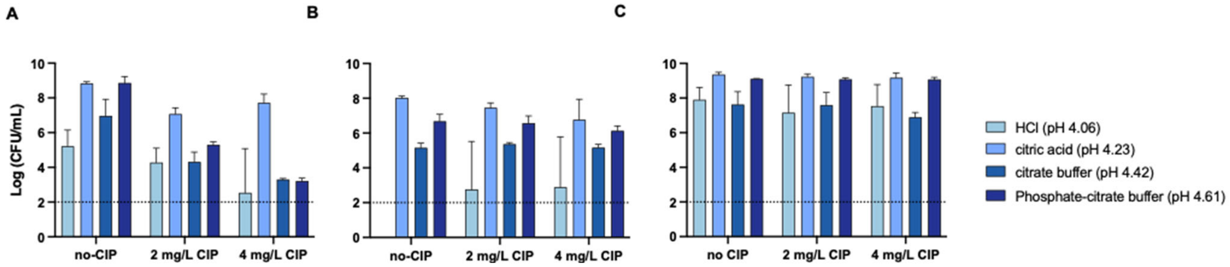

Figure S1. Effects of pH 4, adjusted using HCl, citric acid, citrate buffer, and phosphate buffer, on *P. aeruginosa* growth in ASM after 24 h. (A) PA-Muc and (B) PA-SCV CF isolates and (B) PAI. Each experiment was performed independently at least two times. Data are presented as mean ± SEM. Dash lines indicate the detection limit.

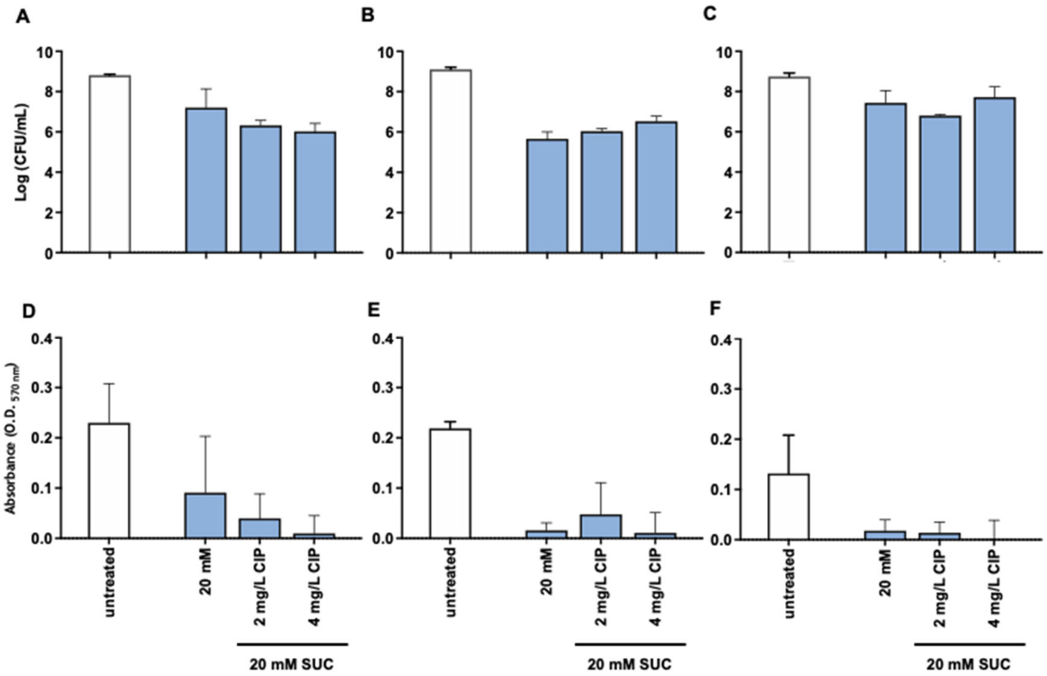

Figure S2. Effect of 20 mM of succinic acid (SUC) activity and its interaction with 2 and 4 mg/L of ciprofloxacin (CIP) against *P. aeruginosa* (A, D) Pa-Muc, (B, E) PA-SCV and (C, F) PAI when applied at 0 h on (A-B) planktonic growth and (E-F) biofilm formation in TSB. All experiments were performed independently at least three times. Values are the mean ± standard deviation.
